# The Interplay of Nitric Oxide and Nitrosative Modifications in Maize: Implications for Aphid Herbivory and Drought Stress

**DOI:** 10.3390/ijms252011280

**Published:** 2024-10-20

**Authors:** Hubert Sytykiewicz, Paweł Czerniewicz, Magdalena Ruszczyńska, Katarzyna Kmieć

**Affiliations:** 1 Institute of Biological Sciences, Faculty of Natural Sciences, University of Siedlce, 14 Prusa St., 08-110 Siedlce, Poland; pawel.czerniewicz@uws.edu.pl (P.C.); mr40@stud.uws.edu.pl (M.R.); 2 Department of Plant Protection, Faculty of Horticulture and Landscape Architecture, University of Life Sciences in Lublin, 7 Leszczyńskiego St., 20-069 Lublin, Poland; katarzyna.kmiec@up.lublin.pl

**Keywords:** nitric oxide, peroxynitrite anion, nitrosative modifications, plant–insect interactions, drought stress, maize, bird cherry-oat aphid

## Abstract

Nitric oxide (NO) and other reactive nitrogen species (RNS) are considered to be signaling molecules in higher plants involved in the regulation of growth and development processes. However, the molecular mechanisms of their formation, removal, and participation in plant responses to adverse environmental stimuli remain largely unclear. Therefore, the aim of this study was to assess the influence of selected single stresses and combined stresses (i.e., *Rhopalosiphum padi* L. aphid infestation, drought, aphid infestation, and drought) and post-stress recovery on the contents of NO and peroxynitrite anion (ONOO^−^), as well as the levels of mRNA and protein nitration (i.e., the 8-nitroguanine and protein 3-nitrotyrosine amounts, respectively), in maize seedlings (*Zea mays* L.). Moreover, the expression patterns of the two tested genes (*nos-ip*, encoding nitric oxide synthase-interacting protein, and *nr1*, encoding nitrate reductase 1) involved in NO metabolism in maize plants were quantified. We identified significant intervarietal, time-course, and stress-dependent differences in the levels of the quantified parameters. Under the investigated stress conditions, the aphid-resistant Waza cv. seedlings were characterized by a higher and earlier NO accumulation and mRNA nitration level and an increased expression of the two target genes (*nos-ip* and *nr1*), compared to the aphid-susceptible Złota Karłowa cv. seedlings. Conversely, the Złota Karłowa plants responded with a greater elevation in the content of ONOO^−^ and protein 3-nitrotyrosine than the Waza cv. plants The multifaceted role of NO and its derivatives in maize plants challenged by single and combined stresses, as well as during post-stress recovery, is discussed.

## 1. Introduction

Maize is one of the most important crop species, along with wheat and rice [1,2]; it provides a source of raw material for the fermentation, paper, and energy industries and is utilized in food production and animal feeding [3,4,5]. Unfortunately, constant changes in climate could cause yield reduction, affecting the productivity of maize [6]. Increased interest in maize in recent years is associated with its high production potential and possibility of cultivation under different and/or unstable climatic conditions [2,3,6]. In addition, maize has been increasingly used as a model organism in a wide array of physiological, biochemical, genetic, and molecular biology studies of plants [7,8,9]. The currently observed climate changes are associated with the extreme environmental factors (e.g., rapid temperature increases, prolonged periods of drought, strong winds, heavy rains, and mechanical damage to the leaf and stem tissues) that exert a stressful impact on plants [10,11,12]. Moreover, abiotic stressors may increase the susceptibility of the cereal plants to various biotic stresses, such as gradations of aphid infestation, which secondarily contribute to the transmission of plant viruses and other phytopathogens [12,13,14]. Environmental stress exposure may lead to deleterious morphological effects and disturbances during physiological processes in plants [8,15,16]. In addition, plants exposed to drought or biotic stresses experience a broad range of metabolic imbalances, including the significant modulation of enzymatic activities as well as profound transcriptional reconfigurations. Several signaling cascades (e.g., mitogen-activated protein kinase (MAPK), Ca^2+^, and abscisic acid (ABA)-mediated pathways) are activated in stressed plants to increase their tolerance to environmental stimuli [2,8,13,14,17]. Drought and cereal aphid infestation may also cause a reactive oxygen species (ROS) overaccumulation, leading to oxidative stress and possible oxidative damage to crucial macromolecules (e.g., nucleic acids, proteins, and lipids) [13,17]. In contrast, it has been shown that in addition to ROS, reactive nitrogen species (RNS) may play a significant role in signaling pathways in plants [18,19]. However, the role of RNS, the nitrosative modifications of nucleic acids and proteins, and the expression of the genes involved in nitric oxide (NO) metabolism in plants exposed to single or combined stresses remain largely unknown.

The purpose of this study was to elucidate the impact of drought and aphid infestation (*Rhopalosiphum padi* L.), as well as the combined (aphid infestation and drought) stress on the contents of NO, peroxynitrite anion (ONOO^−^), and the nitration rate of DNA, mRNA, and proteins in maize (*Zea mays* L.) seedling leaves. Biotests were performed on two maize cultivars (i.e., relatively aphid-resistant Waza and aphid-susceptible Złota Karłowa). It was previously reported that these two maize varieties were characterized by differential accumulation patterns of the oxidative stress markers of crucial macromolecules (such as nucleic acids, proteins, and lipids) in response to aphid infestation [13]. Furthermore, we quantified the expression levels of the two genes (*nos-ip*, encoding nitric oxide synthase-interacting protein, and *nr1*, encoding nitrate reductase 1) involved in NO homeostasis in maize plants. In addition, fluctuations in the content of the quantified parameters in the maize seedlings during post-stress recovery were assessed. This is the first comprehensive report unveiling the variety-dependent mechanisms underlying NO metabolism and the nitrosative modifications of nucleic acids and proteins in maize plants infested with insects and exposed to drought and to the combined (insects and drought) stresses.

## 2. Results

### 2.1. The Impact of Aphid Infestation, Drought, and the Combined Stresses on the Contents of Nitric Oxide (NO) and Peroxynitrite Anion (ONOO^−^) in Z. mays Seedlings

**Figure 1:**
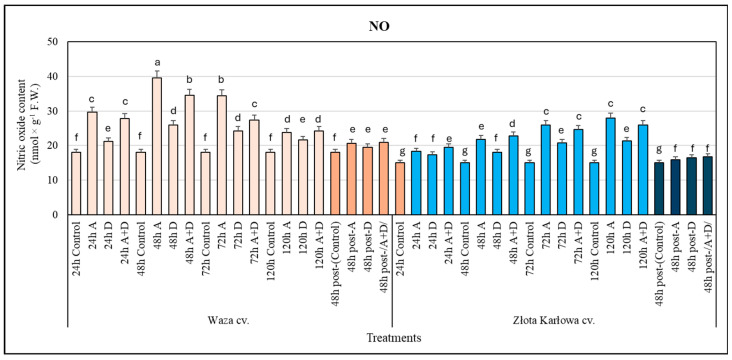
Generation of nitric oxide (NO) in *Z. mays* seedlings treated with the single and combined stresses: Waza cv. (relatively aphid-resistant) and Złota Karłowa cv. (aphid-susceptible). A, aphid infestation; D, drought stress; and A+D, aphid infestation and drought stresses. Control—non-stressed *Z. mays* seedlings. The maize plants were treated with stressing factors for 24 h, 48 h, 72 h, and 120 h. Post-A, post-D, post-/A+D/—the maize seedlings at 48 h after termination of stress exposure. Different letters above the error bars designate significant differences between the mean (±SD) in the NO content in the maize seedlings (Tukey’s test; *p* < 0.01).

The performed biotests uncovered significant differences in the changes in NO and ONOO^−^ content in the seedlings of the two tested maize varieties (i.e., Waza and Złota Karłowa) (Figure 1). The Waza plants were characterized by a significantly higher accumulation of NO at 24–78 h than the Złota Karłowa seedlings, in comparison with the respective controls. In contrast, long-term stress exposure (120 h) led to slightly higher increases in the NO content in the Złota Karłowa seedlings than in the Waza plants (42–87% and 20–35%, respectively) in comparison with the controls. The aphid infestation caused the highest elevation in NO levels in the Waza plants at 48 h and in the Złota Karłowa seedlings at 120 h. Moreover, the three tested types of stressors (A—aphid infestation, D—drought, (A+D)—combined aphid infestation and drought) evoked a gradual increase in the amount of NO in the Złota Karłowa seedlings. However, the drought stress caused lower increments in the NO content in the plants of both examined cultivars, compared with the aphid infestation and the combined (A+D) stresses.

**Figure 2:**
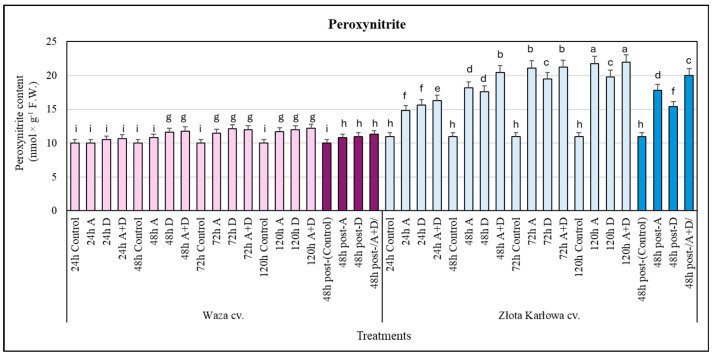
Stress-evoked alternations in the peroxynitrite anion (ONOO^−^) contents in the maize seedlings: Waza cv. (relatively aphid-resistant) and Złota Karłowa cv. (aphid-susceptible). A, aphid infestation; D, drought stress; and A+D, aphid infestation and drought stresses. Control—non-stressed *Z. mays* seedlings. The maize plants were treated with stressing factors for 24 h, 48 h, 72 h, and 120 h. Post-A, post-D, post-/A+D/—the maize seedlings at 48 h after termination of stress exposure. Different letters above the error bars designate significant differences between the mean (±SD) in the ONOO^−^ content in the maize seedlings (Tukey’s test; *p* < 0.01).

Moreover, the ONOO^−^ content in the Waza cv. seedlings treated with the examined stressors was slightly elevated (5–20% increases) compared with the control (Figure 2). In contrast, the Złota Karłowa cv. seedlings exposed to the three studied stresses (A, D, and A+D) were characterized by a gradual increase in the content of ONOO^−^ from 24 h to 120 h. The highest elevations (approx. 2-fold) in the amount of ONOO^−^ were found at 120 h in the Złota Karłowa plants infested with aphids and exposed to the combined (A+D) stress, in relation to the controls. The drought stress induced lower increases (42–80%) in the ONOO^−^ content in the Złota Karłowa seedlings, in comparison with the non-stressed control.

In addition, at 48 h of post-stress recovery (after termination of exposure to the examined stressors), there was evidence of a reduction in the content of NO, reaching similar levels to those of the controls in the seedling leaves of both tested maize cultivars. Moreover, at this time, slightly lower increases in the ONOO^−^ content in the Złota Karłowa seedlings, compared with 120 h of exposure to the stressors, were demonstrated. At 48 h post-stress, the amount of ONOO^−^ in the Waza plants subjected to the three stress types was comparable with, and modestly lower than, that in the seedlings exposed to the examined stressors at 120 h (Figure 1 and Figure 2).

### 2.2. Stress-Stimulated Changes in the Level of Selected Nitrosative Modifications of mRNA, DNA, and Proteins in the Maize Plants

**Figure 3:**
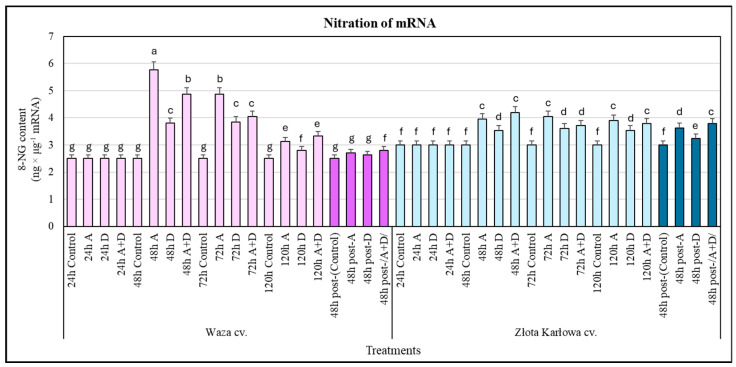
Impact of the investigated stressors on the content of 8-nitroguanine (8-NG) in mRNA in *Z. mays* seedlings: Waza cv. (relatively aphid-resistant) and Złota Karłowa cv. (aphid-susceptible). A, aphid infestation; D, drought stress; and A+D, aphid infestation and drought stresses. Control—non-stressed *Z. mays* seedlings. The maize plants were treated with stressing factors for 24 h, 48 h, 72 h, and 120 h. Post-A, post-D, post-/A+D/—the maize seedlings at 48 h after termination of stress exposure. Different letters above the error bars designate significant differences between the mean (±SD) in the content of 8-NG in mRNA in the maize seedlings (Tukey’s test; *p* < 0.01).

The stressed seedlings of Złota Karłowa and Waza cvs did not respond to changes in the content of 8-nitroguanine (8-NG) in mRNA after 24 h, compared with the controls (Figure 3). However, at 48 h of the biotests, the aphid-infested Waza seedlings were characterized by a significant increase (approx. 2.4-fold) in the content of 8-NG in mRNA, and a slightly lower increase (2-fold) was observed in the Waza cv. seedlings treated with the combined aphid infestation and drought (A+D) stresses, while the lowest increment (1.5-fold) was shown in the drought-exposed plants. At successive intervals of exposure time to the stressors, gradually lower increases in the determined parameter were demonstrated in the Waza seedlings in relation to the control plants. Furthermore, the Złota Karłowa cv. seedlings were characterized by 18–40% elevations (depending on the experimental variant) in the content of 8-NG in mRNA in response to the three stressors studied. In addition, after 48 h of post-stress recovery, the Waza and Złota Karłowa seedlings were characterized by only slightly lower increases in the levels of mRNA nitration in comparison with those in the respective seedlings at 120 h of stressor exposure. It should be noted that the genomic DNA in the stressed maize plants of the two examined cultivars had only slight and insignificant increments (up to 1.2%) in the content of 8-NG, compared with the non-stressed controls.

**Figure 4:**
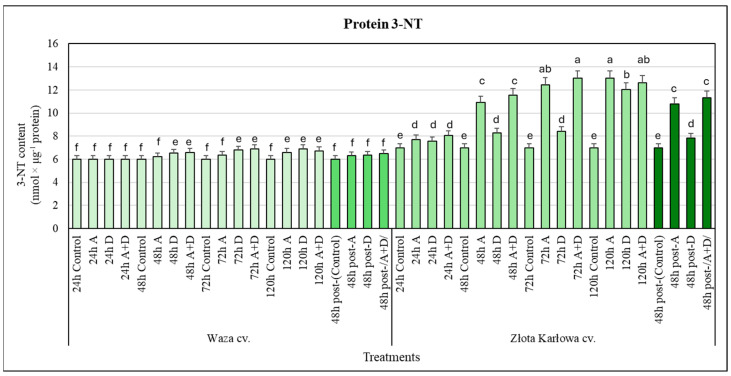
Effect of the examined stresses on the amount of 3-nitrotyrosine (3-NT) in proteins in the maize seedlings: Waza cv. (relatively aphid-resistant) and Złota Karłowa cv. (aphid-susceptible). A, aphid infestation; D, drought stress; and A+D, aphid infestation and drought stresses. Control—non-stressed *Z. mays* seedlings. The maize plants were treated with stressing factors for 24 h, 48 h, 72 h, and 120 h. Post-A, post-D, post-/A+D/—the maize seedlings at 48 h after termination of stress exposure. Different letters above the error bars designate significant differences between the mean (±SD) in the protein 3-NT content in the maize seedlings (Tukey’s test; *p* < 0.01).

Furthermore, the aphid-resistant Waza seedlings responded with only marginal and insignificant increases (5–15%) in the amount of 3-nitrotyrosine (3-NT) in the proteins in response to the three examined stressors (at 48–120 h), compared with the controls (Figure 4). Conversely, the aphid-susceptible Złota Karłowa seedlings responded with gradual increments in the amount of 3-NT in the proteins as a result of exposure to the studied stressors (at 24–120 h). Moreover, the highest increases in the protein nitration levels were noted in the aphid-infested plants at 120 h and in the seedlings under combined (A+D) stress after 72 h and 120 h. Statistically significant lower increases in the content of protein 3-NT in the Złota Karłowa plants exposed to drought (at 48–120 h) were determined. After 48 h of post-stress recovery, the Waza plants maintained low increases (4–8%) in the content of protein 3-NT, which were similar to the levels detected in the seedlings treated with the stressors for 48–120 h. Moreover, the Złota Karłowa plants were characterized by slightly lower elevations in protein nitration, compared with the levels in the seedlings at 120 h of the stressor treatments (Figure 3 and Figure 4).

### 2.3. Stress-Induced Alternations in the Expression Patterns of nos-ip and nr1 Genes in the Maize Seedlings

**Figure 5:**
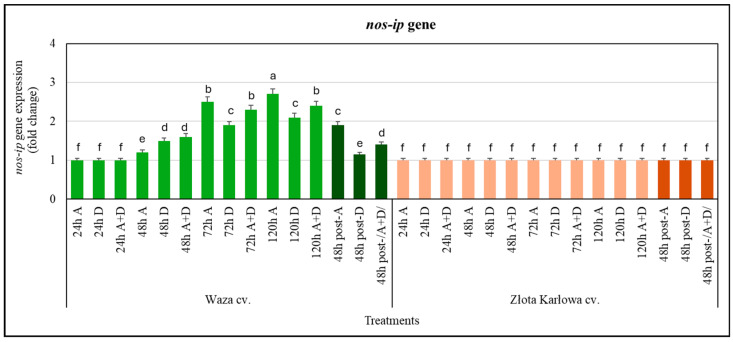
Transcriptional responses of the *nos-ip* gene in *Z. mays* seedlings exposed to the examined abiotic and biotic stressors: Waza cv. (relatively aphid-resistant) and Złota Karłowa cv. (aphid-susceptible). A, aphid infestation; D, drought stress; and A+D, aphid infestation and drought stresses. The maize plants were treated with stressing factors for 24 h, 48 h, 72 h, and 120 h. Post-A, post-D, post-/A+D/—the maize seedlings at 48 h after termination of stress exposure. Data are presented as *n*-fold changes (mean ± SD) in the target gene expression in the stressed plants, compared with the control. Different letters above the error bars designate significant differences in the relative expression levels of the *nos-ip* gene in the maize seedlings (Tukey’s test; *p* < 0.01).

It has been demonstrated that the *nos-ip* gene (encoding nitric oxide synthase-interacting protein) in the Złota Karłowa seedlings was not responsive to the three stresses tested, when compared with the control plants (Figure 5). Conversely, except for the 24 h interval, the Waza plants responded with increases in *nos-ip* gene expression in relation to the stressors examined. Furthermore, there was a gradual increase in the transcriptional activity of the target gene in the seedling leaves of the stressed Waza cv. (from 48 to 120 h). The highest increase (approx. 2.7-fold) in the amount of the tested transcript was recorded at 120 h of aphid infestation. In addition, a slightly lower upregulation of the tested gene was found at 72 h of aphid infestation and at 120 h of exposure to the combined (A+D) stresses (2.5- and 2.4-fold increases, respectively).

**Figure 6:**
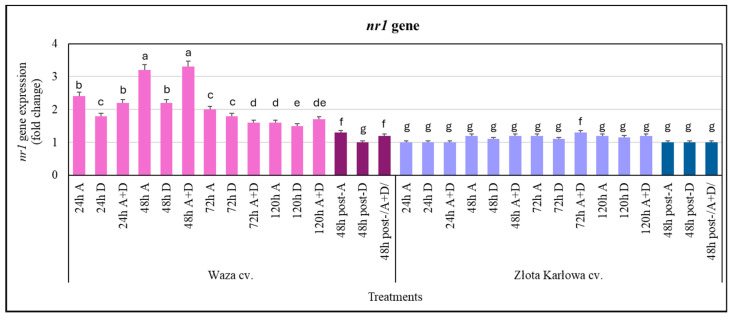
Transcriptional responses of the *nr1* gene in the stressed maize seedlings: Waza cv. (relatively aphid-resistant) and Złota Karłowa cv. (aphid-susceptible). A, aphid infestation; D, drought stress; and A+D, aphid infestation and drought stresses. The maize plants were treated with stressing factors for 24 h, 48 h, 72 h, and 120 h. Post-A, post-D, post-/A+D/—the maize seedlings at 48 h after termination of stress exposure. Data are presented as *n*-fold changes (mean ± SD) in the target gene expression in the stressed plants, compared with the control. Different letters above the error bars designate significant differences in the relative expression levels of *nr1* gene in the maize seedlings (Tukey’s test; *p* < 0.01).

Regarding the *nr1* gene (encoding nitrate reductase 1), the stressed Złota Karłowa seedlings did not respond with changes in its expression after 24 h, and low increases in the transcriptional activity (up to 28%) were found at subsequent intervals of exposure time to the three stressors tested (Figure 6). In contrast, the Waza cv. seedlings were characterized by an increased expression of the *nr1* gene in response to each stress type tested. It was noted that the greatest increase in *nr1* gene expression occurred during the first two intervals of exposure time to the stressors (at 24 h and 48 h). The highest upregulation of this gene was found at 48 h in the aphid-infested seedlings and in the plants treated with the combined (A+D) stresses (3.3- and 3.4-fold increases, respectively). Furthermore, from 72 h to 120 h of stress exposure, progressively lower increases (1.5–2.0-fold) in *nr1* gene expression in the Waza plants were shown, compared with the controls.

It was also demonstrated that, after 48 h of post-stress recovery, the expression of both the *nos-ip* and *nr1* genes was unchanged in the Złota Karłowa seedlings, compared with the controls. The expression levels of the two target genes in the Waza plants were significantly lower than in the seedlings at 120 h of stress exposure. Moreover, the drought-stressed Waza plants at 48 h post-stress had lower elevations in the expression of the two tested genes in relation to the controls, while the highest increases in expression were recorded in the aphid-infested Waza seedlings (Figure 5 and Figure 6).

## 3. Discussion

Although some studies have been conducted on different maize cultivars under drought stress, the molecular mechanisms underlying the adaptation and tolerance to this stressing factor remain unclear [20,21]. Recently, Gu et al. [22] demonstrated the pivotal role of the WRKY transcription factor (encoded by the *ZmWRKY30* gene) in the leaves of a maize transgenic line exposed to drought. Under dehydration conditions, the expression of the *ZmWRKY30* gene increased in the maize in relation to the control. In addition, the mutator-interrupted *ZmWRKY30* homozygous mutant (*zmwrky30*) was more sensitive to drought stress compared with the null segregant. This mutant had a decline in the activity of the antioxidant enzymes (catalase, peroxidase, and superoxide dismutase) and proline content, as well as an increased amount of malondialdehyde (MDA). The biological functions of the *ZmWRKY30* gene and WRKY transcription factors are associated with the regulation of ROS metabolism and the contents of proline and *myo*-inositol [22]. Furthermore, Liu et al. [23] demonstrated that the overexpression of the *ZmHsf28* transcription factor in transgenic maize (*ZmHsf28OE*) increased drought tolerance via stimulation of the abscisic acid (ABA) and jasmonate (JA) signaling pathways and consequently activated the antioxidative mechanisms in the stressed plants.

In general, stress factors affecting plants may cause a variety of detrimental effects (e.g., tissue damage, chlorosis, necrosis, organ deformation, inhibition of growth and development processes, significant disruption of intracellular homeostasis, suppression of cellular respiration and photosynthesis, reduction in the content of chlorophylls, carotenoids, and photoassimilates, disturbances in biogenesis, and turnover of primary and secondary metabolites) [9,15,24,25]. During biotic and abiotic stress conditions, various molecular reactions occur in plants. These include excessive generation of ROS, such as superoxide anion (O_2_^·−^), hydroperoxide (HO_2_^·^), alkoxyl (RO^·^), and hydroxyl (∙OH) radicals, as well as non-radical hydrogen peroxide (H_2_O_2_) and singlet oxygen (^1^O_2_). ROS are mostly generated in chloroplasts, mitochondria, peroxisomes, apoplasts, and plasma membranes [26].

In studies performed on various maize hybrids and inbred lines, wheat, and rice, drought stress increased the ROS content, relative to the controls [17,27]. Sytykiewicz et al. [13] revealed a significant ROS overproduction in maize seedlings (Waza and Złota Karłowa cvs) infested with cereal aphids. Importantly, increased production of ROS contributes to an elevated generation of NO, resulting in the formation of reactive nitrogen species (RNS) [28]. H_2_O_2_ may actively influence NO synthesis via regulation of the nitrate reductase (NR) enzyme activity [29]. ROS and RNS play an active role in molecular feedback in several signaling pathways in plant cells [30]. During environmental stresses, diverse forms of RNS and ROS are produced. Most damaging to plant cells is the reaction of one reactive form with another. For example, when O_2_^·−^ over-reacts with NO, a strong oxidant, ONOO^−^, is formed, which is inherent in the post-translational modification (PTM) of tyrosine nitration in proteins and may also cause nitrosative modifications of nucleic acids and polyunsaturated fatty acids in plants. In addition, ONOO^−^ disrupts NADH-dependent peroxisomal hydroxypyruvate reductase through nitration [31].

Our study unveiled significant differences in the overaccumulation dynamics of both NO and ONOO^−^ between Waza and Złota Karłowa cvs seedlings, compared with the respective controls. The Waza plants reached higher increases in NO content during the early phases of the examined stress exposure (at 24–78 h) than the Złota Karłowa cv. seedlings Furthermore, after 120 h under stress conditions, the Złota Karłowa seedlings contained a slightly greater NO amount in relation to the Waza plants. The opposite trend was demonstrated with regard to the content of ONOO^−^ in the stressed maize plants. The Waza seedlings responded with slight and similar increases in the level of ONOO^−^ under the examined stress treatment, in relation to the controls. However, the stress-exposed Złota Karłowa plants showed a progressively higher increase in ONOO^−^ content (reaching a maximal amount at 120 h) compared with the Waza plants. In addition, the aphid infestation and the combined (A+D) stress induced higher increments in the NO and ONOO^−^ contents in the leaves of both tested maize cultivars in comparison with the effect of drought stress. It has been postulated that ONOO^−^ accumulation in plants may be associated with the regulatory functions of NO availability and activity under both physiological and stressful conditions [32]. Mai et al. [33] found an elevated production of crucial signaling molecules (i.e., jasmonic acid, ethylene, salicylic acid, and NO) in the seedlings of *Pisum sativum* L. exposed to *Acyrthosiphon pisum* (Harris) infestation. According to these authors, the increased NO generation in the infested plants occurred at 48 h, which suggests the involvement of NO in the regulation of tolerance mechanisms.

We found a substantially greater elevation in the nitration level of mRNA in the stressed seedlings of Waza cv. than in Złota Karłowa cv. Furthermore, the genomic DNA in the seedling leaves of both tested maize cultivars exposed to the three types of stresses contained only negligible increases (up to 1.2%) in the content of 8-NG in relation to the controls. The rate of nitration of the DNA, mRNA, and proteins in plants subjected to environmental stressors is poorly understood. The reaction of ONOO^−^ and guanine (mainly at the C8 position) results in the formation of a few nitration products, including 8-nitroguanine (8-NG) [34]. The accumulation of 8-nitroguanine is a well-recognized biomarker of nucleic acid nitration [32]. It has been postulated that DNA nitration is a process that occurs at a much lower intensity than that of RNA nitration. This is due to differences in the structure of the nucleic acids, their stability, and their subcellular localization [34]. The signal report indicated that a fungal infection (*Phytophthora infestans*) caused a significant elevation in mRNA nitration in *Solanum tuberosum* L. leaves in comparison with the non-stressed controls. These increases in nitration levels may be associated with regulatory signaling mechanisms leading to a hypersensitivity response (HR) and programmed cell death triggered by the fungal infection [32,34].

Our results demonstrated very low increases in the protein 3-NT in the leaves of the Waza cv. seedlings in response to the tested stressors. However, the Złota Karłowa cv. plants exposed to these stress factors were characterized by a considerably higher and more progressive accumulation of the protein 3-NT (at 24–120 h), compared with the controls. The content of 3-nitrotyrosine (3-NT) in proteins has been considered a biomarker of nitrosative stress, mainly owing to the irreversible nature of this type of nitration and the synchronous accumulation of this component along with increases in the content of ONOO^−^. However, a few authors emphasized that there is possibly a more complex regulatory effect of the 3-NT amount on the biological activity of proteins in plants [32].

Plant nitrate reductases (NRs) may produce both NO and ONOO^−^ under nitrate availability and aerobic conditions. In addition, some pharmacological studies showed that the generation of NO catalyzed by nitrate reductase was suppressed by sodium azide (NR inhibitor) and a decline in oxygen level [35]. Our results revealed that the transcriptional activity of the *nr1* gene (encoding nitrate reductase 1) was significantly upregulated in the Waza cv. plants (especially at 24–48 h of stress exposure). Conversely, the expression level of this gene in the Złota Karłowa cv. seedlings remained comparable with the control, or only slightly increased. This indicates that Waza cv. has a greater ability to synthesize NO, which possibly causes a stronger stimulation of NO-dependent signaling pathways to activate complex defense mechanisms in the plants. Furthermore, considering the levels of all the determined parameters, it may be assumed that another route of NO biosynthesis occurs in the Złota Karłowa cv. seedlings. Bacteria and animals produce the enzyme nitric oxide synthase (NOS), which catalyzes the biosynthesis of NO from L-arginine [36]. In plants, the gene encoding this enzyme has not been identified to date. However, the occurrence of NOS-like enzymatic activity has been demonstrated in a few plant species [37,38,39,40]. In addition, according to Phillips et al. [37], the suppression of NOS-like activity in maize plants (Border King cv.) significantly downregulated the expression of the genes encoding ascorbate peroxidase and catalase (involved in H_2_O_2_ scavenging), as well as betaine aldehyde dehydrogenase (linked to the biosynthesis of glycine betaine). This indicates the crucial role of NO in the regulation of the antioxidative mechanisms in maize. Nevertheless, in the *Z. mays* genome, a *nos-ip* gene (encoding nitric oxide synthase-interacting protein) that may function as a negative regulator of NOS has been found. We demonstrated that only the aphid-resistant Waza cv. seedlings were characterized by the upregulation of this gene, whereas the seedlings of aphid-susceptible Złota Karłowa cv. did not respond with any alternations in *nos-ip* expression, compared with the control. This suggests the possibility that the Waza cv. seedlings developed more efficient and sophisticated mechanisms to control the NO content, compared with the other maize variety assessed.

We also revealed that, after 48 h of stress recovery, the Waza cv. maize seedlings had lower increases in the determined parameters compared with Złota Karłowa cv. This indicated that the relatively aphid-resistant maize cultivar was characterized by a more precise and coordinated regulation mechanism of NO metabolism than the aphid-susceptible cultivar. This provides a basis for further and more advanced molecular studies to identify all the genes potentially involved in the biogenesis and turnover of NO in maize plants. In addition, an application of a silencing expression of the two target genes (*nr1*, and *nos-ip*) associated with NO homeostasis in maize tissues would allow greater insights into their functioning in seedlings exposed to multifactorial stressors.

## 4. Materials and Methods

### 4.1. Plant Material

The grains of the two examined Polish cultivars of maize (i.e., Waza and Złota Karłowa) were purchased from local companies (W. Legutko, Jutrosin, Poland; PNOS S.A., Ożarów Mazowiecki, Poland). We previously reported that the Waza and Złota Karłowa varieties were relatively aphid-resistant and aphid-susceptible, respectively [41]. All the tested maize grains were surface sterilized and planted in plastic pots in a climatic chamber FITO DUO (Biogenet, Poland), in accordance with the procedure described previously [13].

### 4.2. Aphids

Bird cherry-oat aphid (*Rhopalosiphum padi* L.) apterous females were gathered from plant crops within the Siedlce district in Poland (52°09′54″ N, 22°16′17″ E). The stock of insects was raised on common wheat (Tonacja cv.) for a year. The aphid culture was maintained in a climatic chamber (FITO 1400 DUO; Biogenet, Poland) under controlled environmental conditions [13].

### 4.3. Experimental Design

The biotests were performed on 14-day-old seedlings of the two investigated maize cultivars (i.e., Waza and Złota Karłowa). Healthy seedlings of similar height of each cultivar (*n* = 120) were randomly selected and divided into four groups: (1) control (non-stressed), *n* = 30; (2) infested with 60 *R. padi* adult parthenogenetic females/plant (*n* = 30); (3) exposed to drought (water withholding) (*n* = 30); and (4) infested with 60 *R. padi* aphids/plant and subjected to drought stress (*n* = 30). The control plants and those infested with aphids (single stress) were watered daily. The experiments were performed in three independent repetitions. The impact of the tested stressors on the biochemical parameters and gene expression in the maize seedlings was assayed at 24 h, 48 h, 72 h, and 120 h. Afterwards, the aphids were removed from the plants, and the leaves from the seedlings of each experimental group were collected, pooled, and homogenized in liquid nitrogen using a sterile pestle and mortar. Additional experiments were carried out to evaluate the contents of the determined parameters (i.e., NO, ONOO^−^, and 8-nitroguanine (8-NG) in mRNA and DNA, protein 3-nitrotyrosine (3-NT), and the expression of *nos-ip* and *nr1* genes (encoding nitric oxide synthase-interacting protein and nitrate reductase 1, respectively) in the *Z. mays* leaves during post-stress recovery. In this regard, after termination of the stress treatments (at 120 h), the insects were removed from the plants, and the maize seedlings (*n* = 30) of the four experimental groups described above were grown under non-stressed conditions. After 48 h of post-stress recovery, the leaves were sampled and homogenized in liquid nitrogen.

### 4.4. Determination of Nitric Oxide (NO) and Peroxynitrite Anion (ONOO^−^)

The contents of NO and ONOO^−^ in the maize samples were measured with the application of the Nitric Oxide Assay Kit (catalogue no. ab65328; Abcam, Cambridge, UK) and the Peroxynitrite Assay Kit (catalogue no. ab233469; Abcam, Cambridge, UK), respectively. Absorbance and fluorescence measurements were taken with the use of the Varioskan LUX multimode microplate reader (Thermo Fisher Scientific Inc., Waltham, MA, USA). The amount of NO and ONOO^−^ in the *Z. mays* seedlings samples was expressed as nanomoles per gram of fresh weight.

### 4.5. Isolation and Purification of Genomic DNA (gDNA), Total RNA, and mRNA

Genomic DNA, total RNA, and mRNA from the maize seedlings were extracted, purified, and quantified as described previously [13].

### 4.6. Measurement of 8-Nitroguanine (8-NG) Amount in mRNA and gDNA

The amount of 8-nitroguanine (8-NG) in mRNA and gDNA in the maize seedlings was assayed using the OxiSelect™ Nitrosative DNA/RNA Damage ELISA Kit (catalogue no. STA-825; Cell Biolabs, San Diego, CA, USA). The content of 8-NG in both examined fractions of nucleic acids in the maize samples was expressed in nanograms per microgram of mRNA and gDNA, respectively.

### 4.7. Determination of Protein 3-Nitrotyrosine (3-NT)

The content of protein 3-nitrotyrosine (3-NT) in the maize samples was assessed using the OxiSelect™ Nitrotyrosine ELISA Kit (catalogue no. STA-305; Cell Biolabs, San Diego, CA, USA) and expressed as nanomoles per microgram of protein. Determination of the protein amount in the probes was quantified with the use of Bradford’s method [42].

### 4.8. Gene Expression Quantification

The total RNA isolated from the maize samples was used as the template during cDNA synthesis. This procedure was achieved using the High-Capacity cDNA Reverse Transcription Kit with RNase Inhibitor (catalogue no. 4374966; Thermo Fisher Scientific Inc., Waltham, MA, USA), according to the manufacturer’s instructions. The relative expression of two target genes—*nos-ip* (encoding nitric oxide synthase-interacting protein; GenBank ID: LOC100272289) and *nr1* (encoding nitrate reductase 1; GenBank ID: LOC542278)—in the maize seedlings was estimated using the comparative Ct (ΔΔCt) method [43]. Custom TaqMan Gene Expression Assays (Thermo Fisher Scientific Inc., Waltham, MA, USA) were used during the study (Table 1). The actin-2 gene (GenBank accession no. EU952376.1; ID: LOC100280540) was used as the housekeeping gene [44]. Measurements of the genes’ expression were carried out with the application of the StepOnePlus Real-Time PCR System (Thermo Fisher Scientific Inc., Waltham, MA, USA), in accordance with the procedure described previously [44]. The transcriptional responses of the two examined genes in the stressed maize seedlings were presented as *n*-fold changes in relation to the control (non-stressed) plants.

### 4.9. Statistical Analyses

The data derived from the experiments were presented as the mean (±SD). The biotests were randomized and performed in three independent series of experiments. Measurements of the content of the determined components in the maize samples from a given round of biotests were carried out in at least three biological replicates and the same number of technical replicates. Factorial analysis of variance (ANOVA) with a consecutive post hoc Tukey’s test was performed (*p* < 0.01 was considered as statistically significant). The statistical analyses were performed using STATISTICA v.10 software (StatSoft Inc., Kraków, Poland).

## 5. Conclusions

The performed study identified significant intervarietal differences in the generation dynamics of NO and ONOO^−^, as well as in the rate of the nitrosative modifications of the mRNA and proteins in maize seedlings exposed to the tested single and combined stresses (i.e., drought, aphid infestation, and aphid infestation + drought). In general, the stressed Waza cv. seedlings were characterized by a higher accumulation of both NO content and nitration of mRNA than the Złota Karłowa cv. seedlings. Conversely, the Złota Karłowa cv. plants contained greater amounts of ONOO^−^ and the protein 3-NT compared with the Waza cv. plants. The upregulation of the *nr1* and *nos-ip* genes in the maize seedlings of aphid-resistant Waza cv. was also demonstrated, which indicated the participation of these genes in maintaining NO homeostasis. The study provides an additional research tool that may be used during the identification and selection of maize cultivars exerting an increased tolerance to environmental stresses. Further research should include the use of combined transcriptomic (mRNA and microRNA) and proteomic analyses, as well as advanced genetic engineering procedures (e.g., gene silencing). Collectively, these would provide greater insights into the highly complex NO metabolism and nitrosative modifications in stressed maize plants.

## Figures and Tables

**Table 1:** List of primers and TaqMan fluorescent probes used for quantification of transcriptional activity of the two target genes in the maize seedlings.

Tested Gene	GenBank Reference Sequence	GenBank Gene ID	Sequences of Primers and TaqMan Fluorescent Probes
*nos-ip* (encoding nitric oxide synthase-interacting protein)	NM_001146776.2	LOC100272289	F: AGCGTTCTCTGTGTCTGTTC R: TTTTACCTATCTCAACGCCCC P: CCTCGGTTTCCTGAAGCACCTGG
*nr1* (encoding nitrate reductase 1)	NM_001305856.1	LOC542278	F: CCATCAACAGCATTACCACAC R: CACACCAGCCATGTCTCG P: TCCAGCGTCACCTCCACCC

F—forward primer; R—reverse primer; P—TaqMan fluorescent probe. Sequences of primers and TaqMan fluorescent probe were designed using Primer Express™ Software v3.0.1 (Applied Biosystems, Waltham, MA, USA).

## Data Availability

The data presented in the study are available in the article.
